# Ultrasound Evidence of Early Fetal Growth Restriction after Maternal Malaria Infection

**DOI:** 10.1371/journal.pone.0031411

**Published:** 2012-02-09

**Authors:** Marcus J. Rijken, Aris T. Papageorghiou, Supan Thiptharakun, Suporn Kiricharoen, Saw Lu Mu Dwell, Jacher Wiladphaingern, Mupawjay Pimanpanarak, Stephen H. Kennedy, François Nosten, Rose McGready

**Affiliations:** 1 Shoklo Malaria Research Unit, Mae Sot, Tak, Thailand; 2 Nuffield Department of Obstetrics and Gynaecology, and Oxford Maternal and Perinatal Health Institute – Green Templeton College, University of Oxford, Oxford, United Kingdom; 3 Mahidol-Oxford Tropical Medicine Research Unit, Mahidol University, Bangkok, Thailand; 4 Centre for Tropical Medicine, Nuffield Department of Clinical Medicine, University of Oxford, Oxford, United Kingdom; University of Copenhagen, Denmark

## Abstract

**Background:**

Intermittent preventive treatment (IPT), the main strategy to prevent malaria and reduce anaemia and low birthweight, focuses on the second half of pregnancy. However, intrauterine growth restriction may occur earlier in pregnancy. The aim of this study was to measure the effects of malaria in the first half of pregnancy by comparing the fetal biparietal diameter (BPD) of infected and uninfected women whose pregnancies had been accurately dated by crown rump length (CRL) before 14 weeks of gestation.

**Methodology/Principal Findings:**

In 3,779 women living on the Thai-Myanmar border who delivered a normal singleton live born baby between 2001–10 and who had gestational age estimated by CRL measurement <14 weeks, the observed and expected BPD z-scores (<24 weeks) in pregnancies that were (n = 336) and were not (n = 3,443) complicated by malaria between the two scans were compared. The mean (standard deviation) fetal BPD z-scores in women with *Plasmodium (P) falciparum* and/or *P.vivax* malaria infections were significantly lower than in non-infected pregnancies; −0.57 (1.13) versus −0.10 (1.17), p<0.001. Even a single or an asymptomatic malaria episode resulted in a significantly lower z-score. Fetal female sex (p<0.001) and low body mass index (p = 0.01) were also independently associated with a smaller BPD in multivariate analysis.

**Conclusions/Significance:**

Despite early treatment in all positive women, one or more (a)symptomatic *P.falciparum* or *P.vivax* malaria infections in the first half of pregnancy result in a smaller than expected mid-trimester fetal head diameter. Strategies to prevent malaria in pregnancy should include early pregnancy.

## Introduction

Malaria remains one of the most common parasitic infection of human pregnancy [1]–[4], and it lowers birthweight whether or not maternal symptoms are present [5]. Even a single episode of treated *Plasmodium (P.) falciparum* or *P.vivax* malaria during pregnancy has a negative effect on birthweight [6], [7]. The mechanisms of this reduction in birthweight include placental insufficiency by sequestration of malaria parasites leading to intrauterine growth restriction (IUGR), premature labour or a combination of the two [8], [9]. The evidence is less clear in *P.vivax* infected pregnancies where placental sequestration is probably limited [10]. Difficulties in estimating gestational age (GA) accurately and diagnosing malaria infection in early pregnancy have complicated the interpretation of previous malaria studies on fetal growth [9], [11]. IUGR may start in the first trimester and influence late pregnancy outcomes [12]. Early antenatal ultrasound - which is essential to date pregnancy accurately [13] - is becoming available in developing countries [14]–[16]. The aim of this study was to assess whether malaria infection affects early fetal growth by comparing the fetal biparietal diameter (BPD) before 24 weeks gestation in infected and uninfected women whose pregnancies had been accurately dated by crown rump length (CRL) measurement before 14 weeks.

## Methods

### Study site and population

The Shoklo Malaria Research Unit (SMRU) is located on the border between Thailand and Burma in Tak province where the majority of people belongs to the Karen ethnic group [17]. *P.falciparum* [18] and *P.vivax* [7] transmissions are low and seasonal [19]. Since 1986 the SMRU has offered free antenatal care (ANC) to refugees and later (1998) to migrant women, including weekly malaria screening to detect and treat all parasitaemic episodes during pregnancy in order to prevent maternal death [18]. There is no presumptive treatment of malaria, chemoprophylaxis or intermittent preventive treatment in pregnancy due to drug resistance. Since the inception of this ANC program, all pregnant women have been encouraged to attend as early as possible in the first trimester. In 2001 antenatal ultrasound was introduced to improve pregnancy dating in this population because of low literacy rates and poor recall of the date of the last menstrual period [14], [20]. At enrolment in the antenatal clinic all women are interviewed for demographics and have anthropometric measurements taken. At every visit ferrous sulphate (200 mg daily) and folic acid (5 mg weekly) for anemia prophylaxis and thiamine (Vitamin B1, 100 mg daily) to prevent infant mortality from beri-beri [21] are offered to all women. Anaemic women receive treatment doses of ferrous sulphate (200 mg three times daily) and folic acid (5 mg daily). All medical and obstetric problems in pregnancy are investigated and treated free of charge by locally trained health workers working in SMRU facilities [22], [23].

### Pregnancy ultrasound

Locally trained health workers (10 sonographers at 3 clinics) obtain all ultrasound scans using Toshiba Powervision 7000 (since 2006), Dynamic Imaging (since 2001), Fukuda Denshi UF 4100 (since 2002) ultrasound machines. Their practice is supervised by doctors certified in fetal ultrasonography. All women are offered two scans in pregnancy. The 1^st^ scan occurs at the booking visit (between 8–14 weeks gestation) where ultrasound is used to determine viability, identify multiple pregnancy and estimate GA by CRL measurement. Based on this GA estimate women then return for a 2^nd^ scan performed at 18–24 weeks to re-assess viability, measure fetal biometry, identify major fetal abnormalities and determine placental location. Fetal biometry is routinely measured twice in each woman at each scan, as part of an existing quality control [14].

The training manual and protocol for obtaining trans-abdominal CRL and biometry measurements were from the British Medical Ultrasound Society recommendation [24].

The BPD is measured at the cross-sectional view of the fetal head at the level of the ventricles by placing the calipers on the outer border of the upper and the inner border of the lower parietal bones (‘outer to inner’, BPD) across the widest part of the skull. Importantly for this study, operators taking fetal measurements were not aware of the maternal malaria status.

### Outcomes and SMRU delivery rooms

All women are encouraged to deliver in the SMRU facilities under supervision of locally trained midwives who speak their language. Women requiring Caesarean section are referred to the nearest Thai hospital; in this study the Caesarean section rate was 3.4% (129/3,779). Each baby is weighed within 24 hours on electronic SECA (Model 336 or 376, accuracy = 10 g) digital newborn scales.

### Ethics statement

This is a retrospective hospital record analysis. For those patients in trials a written informed consent was obtained including storing of data and samples. For the women seen in the ANC the routine clinical records were anonymized and these pregnancy records have been routinely entered into a database since 1987. Permission was granted by Oxford Tropical Research Ethics Committee (reference: OXTREC 28–09) to use these records for analysis.

### Diagnosis of malaria

Malaria is diagnosed by Giemsa stained thick and thin blood films; 200 fields on the thick film are read before being declared negative. All parasite densities are counted per 500 white blood cells or per 1000 red blood cells. *P. vivax* or *P. falciparum* malaria infection is defined by the presence of asexual stages of the respective parasite in the peripheral blood.

### Definitions

Severe malaria is defined as per WHO treatment guidelines [25] and hyperparasitaemic malaria by the presence of at least 4% infected red blood cells in the absence of other signs of severity. Anaemia is defined by a haematocrit less than 30%. Symptomatic malaria is defined by a temperature ≥37.5°C or a history of fever [25]. When a women had at least one symptomatic episode between the 1^st^ and the 2^nd^ scan she was classified as symptomatic. Mid Upper Arm Circumference (MUAC) was measured at the first ANC consultation on an unclothed left arm with a SECA measuring tape (model 212) accurate to one mm and low MUAC is defined as <21.0 cm [26]. Maternal height is measured at the first ANC consultation and short stature is defined as <145 cm. Maternal weight of women wearing the lightest possible clothing, is measured at the first consultation and at the time of the biometry ultrasound scan on mechanical SECA weight scales (model 762) with graduation of 500 grams. Weight gain is defined as the difference in maternal weight between the two scans. The weight in the first trimester is used to calculate the body mass index (BMI): a BMI of <18.5 kg/m2 is considered underweight [27]. Pregnancy duration is defined as 280 days post menstruation. Miscarriage is a pregnancy ending before 28 weeks GA and stillbirth a delivery from 28 weeks or ≥800 g birthweight in which the infant displayed no sign of life (gasping, muscular activity, cardiac activity). The 28-week GA, rather than the current WHO 22-week GA cut-off was chosen, as no infant ventilatory support is available in the clinics. This cut-off has been in place since SMRU was established as the lower limit of viability in this area. Congenital abnormality is considered if any major abnormality was present at birth by staff trained in examination of the newborn.

### Inclusion and Exclusion criteria

**Figure 1:**
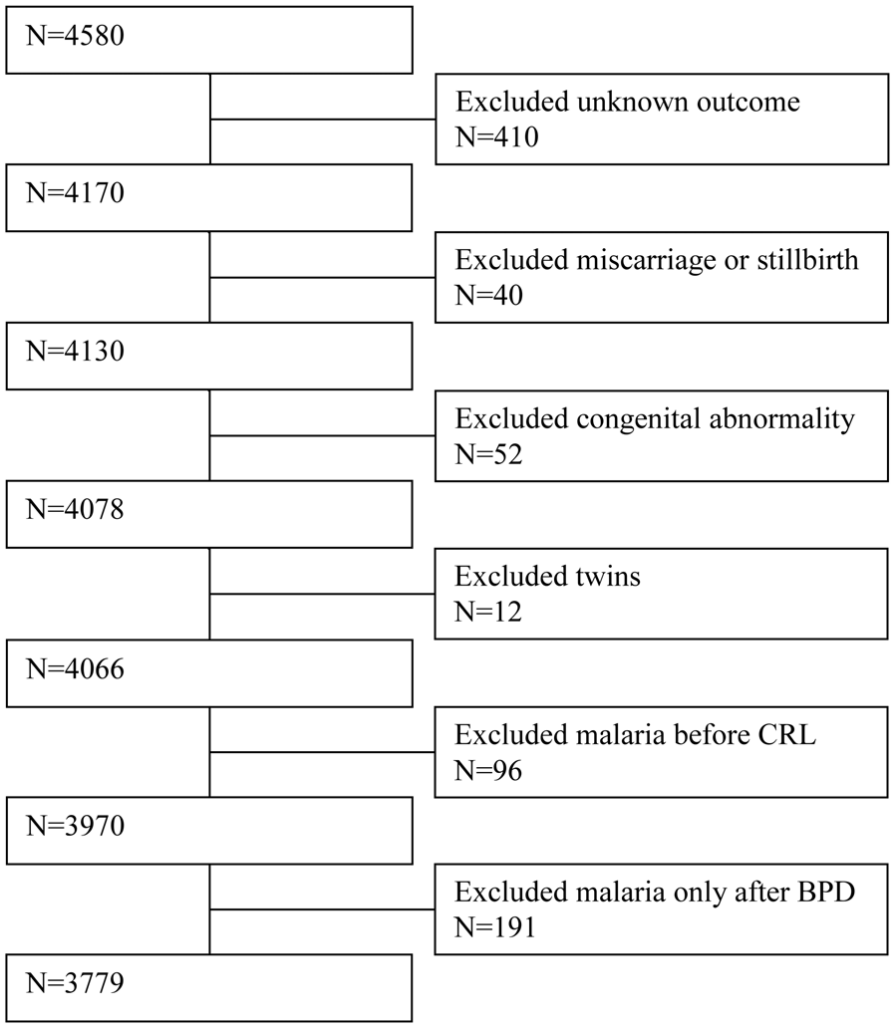
Selection of pregnant women.

All women who had GA estimated by CRL measurement <14 weeks (1^st^ scan) and BPD measured <24 weeks (2^nd^ scan), were included in the analysis. Twin pregnancies, pregnancies that were complicated by miscarriage, stillbirth or fetal structural abnormalities and pregnancies with an unknown outcome were excluded (Figure 1). Women who had their first malaria episode before or at the time of the 1^st^ scan or after the 2^nd^ scan were also excluded. Therefore in this analysis all malaria infected women had their first malaria episode between the first and the second ultrasound scans. For the analysis of birth outcomes women who had another episode after the second scan were excluded to avoid the confounding effects of malaria outside the window of measurement (14^+0^–24^+0^ weeks).

### Statistical Analysis

Clinical data and the results of the two ultrasound scans were entered into a Microsoft Access database and analyzed using SPSS version 18 for Windows. Student's t-test and Mann-Whitney test were used for comparison of means and ranks respectively. Categorical data were compared using the chi-squared test or the Fisher's exact test, as appropriate. The mean of two CRL measurements was used to calculate GA in days using a well established formula [28]. The average of two BPD measurements was recorded as ‘BPD observed’. For the purpose of this study the ‘expected BPD size’ for each GA was calculated using a reference equation for the Karen population [29]. For validation purposes all analyses were repeated for z-scores derived from a chart based on an ethnic Chinese population [30].

Each observed BPD was then converted into a z-score using the following formula:
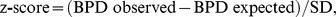
where SD is the standard deviation of the BPD at that GA. The advantage of converting measurements into z scores is that it eliminates variability by GA allowing measurements to be compared [31], [32]. A positive z-score indicates a larger and a negative value a smaller than expected BPD.

In order to assess the impact of malaria infection on fetal growth during early pregnancy, the mean BPD z-scores were compared in women who did and did not have peripheral parasitaemia in the window between the 1^st^ and 2^nd^ scans. Other factors associated with BPD were evaluated by univariate analysis; variables with a significance of P<0.1 were kept in the multivariate regression model. A secondary endpoint was the effect of malaria infection between the 1^st^ and 2^nd^ scan only, on GA and birthweight at delivery. For this purpose a population birthweight centiles chart (per gestational age) was computed from all pregnancies in SMRU with accurate ultrasound dating. Only newborns who were weighed within the first 24 hours after delivery were included [11].

## Results

Between September 2001 and January 2010, 4,580 women had a 1^st^ (CRL measurement <14 weeks) and a 2^nd^ scan (BPD measurement <24 weeks). Women with an unknown pregnancy outcome (410, 9.0%) were excluded from further analysis; they were more likely to be younger and primigravid, to book at a lower GA and have malaria (data not shown). A further 391 women (8.5%) were excluded for miscarriage/stillbirth (n = 40), congenital abnormality (n = 52), twin pregnancy (n = 12), first malaria before the CRL dating scan (n = 96) or first malaria infection after the BPD scan (n = 191) (Figure 1). There were three maternal deaths: two women died from post-partum hemorrhage after delivering stillborn infants (excluded from analysis due to stillbirth); one woman died of severe malaria five weeks post-partum and was included as the infant was live born. Therefore, a total of 3,779 women remained for analysis.

### Frequent intermittent malaria screening in the ANC

The median number of antenatal visits was 23 [range 3–38]. At each visit a malaria smear was obtained and malaria parasites were detected in 930 (1.1%) of the available 86,416 blood slides; these were from 336/3,779 (8.9%) women. The median number of malaria episodes per woman was 2 [range 1–8] for the duration of pregnancy.

### Timing of Malaria episodes

The 336 women who had their first malaria infection in the window between the two scans had a lower BMI, a lower Hct and were younger and more likely to smoke, than the 3,443 uninfected women (Table 1). Of the 336 women with malaria, 240 (71.4%) had only *P.vivax* infections and 96 (28.6%) had *P.falciparum* or both infections (Table 2). In 233 (69.3%) there was a single malaria infection in the window between the two scans; of these 80.3% (187/233) were *P.vivax*. Multiple malaria infections [range 2–4] in the window were diagnosed in 103 women (30.7%). There were only six women (1.8%) with a hyperparasitaemic *P.falciparum* infection in the window and no severe malaria infections (Table 2). Only 114 (33.9%) women had all their episodes of malaria between the two scans and none after the second scan.

**Table 1:** Demographics of the refugee and migrant women from Thai Burmese border, 2001–2010.

	Malaria+	No Malaria	P value
	n = 336	n = 3,443	
Age, years	24.0 20–30	26.0 21–31	**0.006**
Gravida	3 1–4	3 2–4	0.187
Parity	1 [0–3]	1 [0–3]	**0.036**
Nulliparous, % (n)	28.3 (95)	24.3 (836)	0.105
Height, cm$	151 [148–155]	151 [147–155]	0.349
BMI, kg/m^2^ $	20.0 [18.6–21.5]	20.5 [19.0–22.4]	**<0.001**
Weight gain, kg*	2.0 [1.0–3.0]	2.0 [1.0–3.0]	0.265
Hct, %	28 (26–30)	30 (28–32)	<0.001
MUAC, cm#	24.3 [23.0–26.0]	24.0 [22.8–26.0]	0.483
Smoker, % (n)	30.7 (103)	22.5 (773)	**0.001**
NOC during pregnancy	23 19–28	23 17–29	0.485

Median [IQR], or as indicated.

BMI body mass index, Hct Haematocrit at first consultation, MUAC middle upper arm circumference, NOC number of consultations.

+ between the 1^st^ and 2^nd^ scans.

*Weight gain from the first to the second scan; available from 301 in malaria and 2,677 in no malaria group.

# Available from 292 in malaria and 2,626 in no malaria group.

$ Available from 314 in malaria and 3,044 in no malaria group.

**Table 2:** Species, episodes and severity of malaria and mean BPD z-score of women infected between the first and second scan.

	Total malaria episodes	*P.falciparum* or mixed	*P.vivax*	Proportion of women with one episode	Proportion women at least one symptomatic episode	Proportion at least one hyper/severe episode
	N = 336	N = 96	N = 240	N = 336	N = 334#	N = 336
Between 1^st^ and 2^nd^ scan	1 1–4	1 1–3	1 1–4	69.3% (233)	49.1% (165)	1.8% (6)
Mean BPD z-score	−0.57 (1.1)	−0.45 (1.2)	−0.62 (1.1)	−0.51 (1.2)	−0.59 (1.1)	n.a.

Median [min-max], or mean (SD). BPD biparietal diameter; hyper = hyperparasitaemia (≥4% red blood cells infected), n.a. not applicable, P plasmodium.

# Missing data n = 2.

### Effect of malaria on BPD

**Figure 2:**
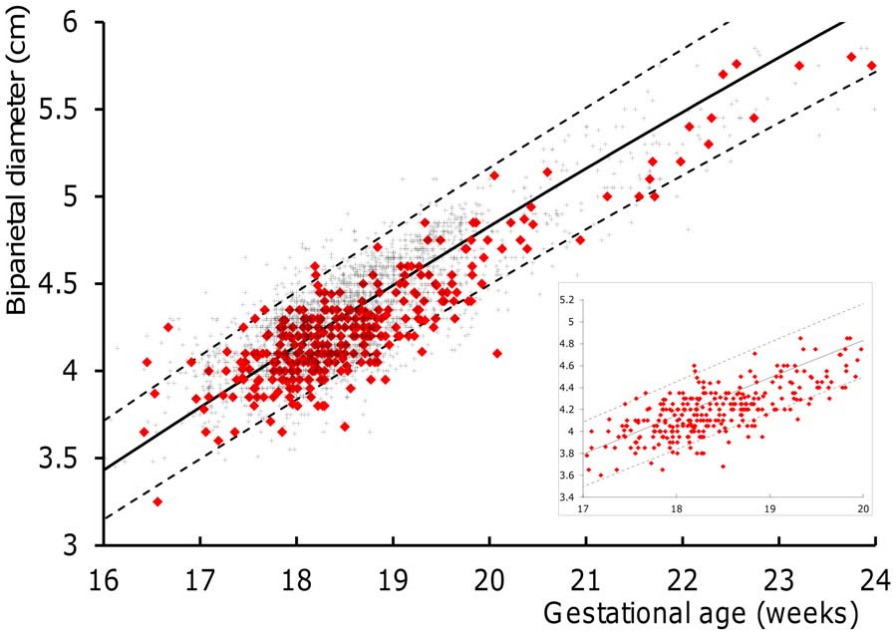
Fetal biparietal diameter measurements in Burmese and Karen pregnant women with and without malaria. The x-axis shows the gestational age (GA) in weeks, based on first trimester dated pregnancies on the Thai-Burmese border from 2001 to 2010. The y-axis depicts the fetal biparietal diameter measurement (BPD) in centimeters. The fetal BPD in pregnant women with malaria (red diamonds, n = 336) and in women without malaria (**+**, n = 3,443) between 16 and 24 GA weeks were superimposed on the 2.5^th^, 50^th^ and 97.5^th^ centiles of a reference equation for this population [29]. Note that the majority of fetal BPD measurements in malaria infected women lie below the 50^th^ centile in both the main figure (16 to 24 GA weeks) and in the inset (17 to 20 GA weeks, where 90% (302/336) of the measurements in malaria infected women were obtained).

**Figure 3:**
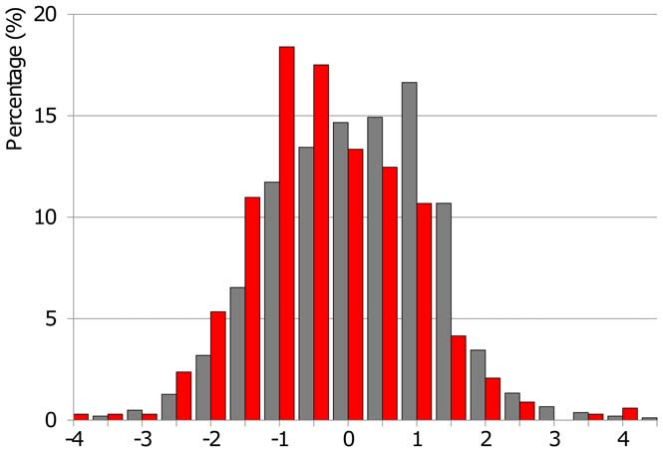
Z-scores of fetal biparietal diameter in Burmese and Karen pregnant women with and without malaria. The x-axis shows the z-score and the y-axis depicts the distribution in percentages. The distribution of z-scores of fetal biparietal diameter in pregnant women with malaria (n = 336, red bars) is significantly lower than in women without malaria (n = 3,443, grey bars) on the Thai-Burmese border from 2001 to 2010.

The BPD measurements of malaria infected and uninfected women were superimposed on the 2.5^th^, 50^th^, and 97.5^th^ centiles of the reference equation [29] and presented graphically between 16 and 24 weeks GA to allow visual comparison (Figure 2). Most of the BPD measurements of women with malaria (red diamonds) were below the 50^th^ centile (Figure 2 and insert, which shows the BPD measurements between 17 and 20 weeks GA in which 90% (302/336) of the measurements were obtained). The BPD measurements were then converted into z-scores that were normally distributed. There was a statistically significant difference between the mean (SD) BPD z-scores of the fetuses of infected and uninfected women (−0.57 (1.13) versus −0.10 (1.17), p<0.001) (Figure 3, Table 3). Both *P.falciparum* (or mixed) (n = 96) and *P.vivax* (n = 240) were associated with a lower mean z-score than uninfected women (−0.45 (1.2), p = 0.006 and −0.62 (1.1) respectively, p<0.001). There was no significant difference in mean (SD) z-scores between women with symptomatic (n = 165) or asymptomatic malaria (n = 169) episodes: −0.59 (1.1) versus −0.54 (1.2) respectively, p = 0.68. Women with multiple malaria infections (n = 103) had the lowest mean (SD) z-scores (−0.72 (1.0), p<0.001) but even a single malaria episode between the 2 scans (n = 233) resulted in a significantly lower mean (SD) BPD z-score: −0.51 (1.2), p<0.001). There was no significant difference in mean z-scores between women with uncomplicated and hyperparasitaemic malaria, but the number of hyperparasitaemic women was too small for analysis (Table 2).

**Table 3:** Risk factors associated with mean BPD z-score as a measure of early fetal growth restriction.

			Univariate		Multivariate (n = 2972)	
		Frequency (%)	Coefficient (95% CI)	P-value	Coefficient (95%CI)	P-value
Teenager	No	3,210 (84.9)				
	Yes	569 (15.1)	−0.02 (−0.13, 0.08)	0.66	*	NS
Primigravida	No	2,848 (75.4)				
	Yes	931 (24.6)	0.07 (−0.02, 0.16)	0.10	*	NS
Smoking	No	2,893 (76.8)				
	Yes	876 (23.2)	0.04 (−0.05, 0.12)	0.43	*	NS
Low MUAC	No	2,801 (96.0)				
	Yes	116 (4.0)	0.07 (−0.14, 0.29)	0.51	*	NS
Short	No	2,995 (89.2)				
	Yes	362 (10.8)	0.04 (−0.09, 0.16)	0.58	*	NS
Low BMI	No	2,738 (81.6)				
	Yes	619 (18.4)	0.18 (0.08, 0.28)	**0.001**	0.15 (0.04, 0.26)	**0.005**
Weight loss	No	2,396 (80.5)				
	Yes	581 (19.5)	0.09 (−0.02, 0.19)	0.10	0.11 (0.00, 0.21)	**0.05**
Anaemia	No	2152 (56.9)				
	Yes	1627 (43.1)	0.16 (0.09, 0.24)	**<0.001**	$	
Malaria	No	3,443 (91.1)				
	Yes	336 (8.9)	0.47 (0.34, 0.60)	**<0.001**	0.50 (0.36, 0.63)	**<0.001**
Symptomatic malaria	No	169 (50.6)				
	Yes	165 (49.4)	0.05 (−0.19, 0.30)	0.68	*	NS
Newborn gender	M	1,936 (51.2)				
	F	1,843 (48.8)	0.25 (0.18, 0.33)	**<0.001**	0.25 (0.17,0.34)	**<0.001**

Significant differences are shown in bold.

BMI body mass index, MUAC middle upper arm circumference, NS not significant.

*Not included in the multivariate analysis.

$ Anaemia was highly collinear with malaria, and the co-variates in the multivariate model were unchanged when anaemia was adjusted for or not.

Aside from malaria, female sex of the fetus, maternal anaemia and low maternal BMI were also significantly associated with a lower mean z-score in the univariate analysis. These remained independent risk factors in the linear regression model which included 2972 women, the remainder being excluded because of missing values mainly maternal weight at the second scan (Table 3). This significantly lower mean BPD z-score (difference in z-score of 0.47 SD) means that malaria during the period between the two scans reduces the diameter of the fetal head by approximately 1 mm when measured at a GA of 22 weeks. The same analysis was repeated for z-scores derived from a Chinese population and except for a small shift in mean z-score (as expected for different populations) the magnitude and significance of the differences were similar (Supporting File S1 and Supporting Table S1).

### Effect of malaria on birth outcomes

Of the 3,083 live born congenitally normal singleton infants weighed within 24 hours of delivery, the z-score for BPD was positively correlated with birthweight centile: B = 0.168 (95% CI 0.128–0.209), p<0.001. Seventy two of the 114 women (63.2%) who had all their malaria infections between the two scans only, had their baby weighted within 24 hours. Neonates from these pregnancies had similar birth outcomes as the ones from uninfected pregnancies (n = 3,011): mean (SD) birthweight 2919 (SD 533) g versus 2973 (SD 433) g, p = 0.40, mean GA 272 (SD 13) days (or 38.9 (SD 1.9) weeks) versus 273 (SD 11) days (or 39.0 (SD 1.6) weeks), p = 0.22 and birthweight centile 0.00 (SD 1.2) versus 0.03 (SD 1.0) respectively. Within this small malaria sub-group (n = 72) there was no association between BPD z-score and birthweight centile.

## Discussion

In this study a significantly smaller fetal BPD was observed by ultrasound when malaria infection occurred in the first half of pregnancy, compared to pregnancies unaffected by malaria. Previous studies have shown that malaria early in pregnancy has an impact on birthweight, but they were limited by small numbers of first trimester exposures [33] or by inaccuracy in the dating of gestation [34]. In this analysis the women had a documented and treated episode of malaria during a specific period between two ultrasound scans. By studying this selected group the effects of malaria on fetal growth could be quantified *in-utero*. Even a single infection of treated *P.vivax* or *P.falciparum* was associated with reduced BPD irrespective of whether the woman was symptomatic or not. The mechanisms underlying the adverse effects of malaria in pregnancy are not fully understood [7], [35], [36]. *P.falciparum* is thought to sequester in the placenta and interfere with materno-fetal exchanges but other mechanisms may also be involved [8], [36]. Systemic or hormonal mechanisms may play a role in *P. vivax* related growth restriction, as there is little evidence that *P.vivax* sequesters in the placenta, like *P.falciparum* does [7], [10], [35], [36]. In non-malaria endemic areas, early pregnancy growth restriction has been associated with miscarriage [37], maternal physical characteristics and lifestyle habits related to early fetal growth [38], and low BPD growth rates between the first and second trimester are associated with increased perinatal mortality and IUGR [39], [40]. In malaria endemic areas ultrasound studies have related malaria in pregnancy to changes in maternal and fetal blood flow [41], [42] and associated malnutrition and malaria in pregnancy with IUGR [43]. So the results presented here are not entirely unexpected but show for the first time the direct evidence of the effect of malaria (both falciparum and vivax) on fetal growth. More surprising perhaps is that low BPD growth was observed after a single (even asymptomatic) infection and despite early treatment.

Studies on the impact of malaria in pregnancy have almost always focused on birthweight. However, for infections that occur in early pregnancy, the size of the fetal head may be a more appropriate indicator of growth restriction. It has been shown that the growth velocity of the fetal head (in mm/day) is maximal during the second trimester [44]–[46]. In contrast the fetal “weight velocity” (in g/week) peaks in the third trimester. This weight gain velocity curve has often been cited wrongly as a fetal “growth velocity” curve [47], [48]. The characteristics of the “fetal head size” and “fetal weight” growth velocity curves are similar but the timing is different. One of the strengths of this study is that the timing of the BPD measurement coincided with the maximal growth velocity of the fetal head, making it a better marker of the effect of malaria in early pregnancy than birthweight.

The reduction in BPD size occurred despite prompt treatment with effective antimalarials in this setting, which highlights the importance of prevention in pregnancy. Multiple episodes of *P.vivax* are most likely from liver stage relapses instead of newly acquired infections. There is no treatment available in pregnancy for liver stages. Furthermore, in this setting of multidrug resistant parasites there are no safe and effective drugs available to prevent malaria from the start of pregnancy. To protect the developing fetus from growth restriction from both symptomatic and asymptomatic *P.vivax* and *P.falciparum* infections, prevention strategies from early pregnancy onwards or even pre-pregnancy interventions should be considered [33], [34], [49], [50].

There are some limitations to this analysis. Firstly, although dating by CRL is generally considered to be the most accurate method of estimating GA, some factors that may have had an impact on CRL dating, for example maternal age [51] or haematocrit [38]. Such factors are difficult to control for in this type of population because the date of the last menstrual period is often not available. The second limitation is that the observed difference in BPD is small and within the range of error for most ultrasound machines and sonographers. The scans were obtained by locally trained technicians who were previously reported to have a mean difference of 0.43 (SD 1.21) mm in their BPD measurements in scans between 18 and 24 weeks, corresponding to 0.12 (SD 0.36) weeks [14]. The observed reduction in BPD in malaria infected pregnancies at 22 weeks is within the range of this measurement error. However the data of this retrospective analysis were derived from the entire population of women attending ANC for their routine ultrasound scans, which minimizes selection bias. In addition the sonographers were not aware whether women had malaria or not during pregnancy and therefore observer bias is unlikely. If there was no true effect of malaria infection on BPD any observed difference would likely be occulted by the expected measurement error of the examiners. In contrast, within this large population of routine ultrasound scans, malaria in the late first and/or second trimester was the largest risk factor for a smaller BPD. Thirdly, in order to examine the effect of the malaria between the scans on birth outcomes, a highly selected group of women was studied for this analysis. This small group (N = 72) had their malaria infections only between the two scans, and was malaria free in the time period where weight gain velocity is highest. Therefore no firm conclusions on the relationship between malaria in early pregnancy, its impact on BPD and birth outcome can be drawn from this analysis. Finally, other biometric parameters, such as fetal head circumference, were not available for most women.

Intermittent preventive treatment, one of the main WHO recommended strategies for malaria prevention and control during pregnancy in areas of stable malaria transmission, aims to provide two or three treatment doses after quickening (around 20 weeks GA) at least one month apart [48]. This approach fails to protect women in the gestational weeks of the highest fetal head growth velocity. Fetal growth has been postulated to be a dynamic system where pulsatile characteristics of saltatory growth events are able to change throughout pregnancy [52]–[55]. Such pulsatile growth events all the way through pregnancy stress the importance of protecting each fetus from the effects of malaria parasites starting as early as possible in pregnancy.

## Supporting Information

File S1 Calculations derived from the equations based on a Chinese population.
(DOC)

Table S1 Factors associated with mean BPD z-score when calculated from the Chinese equation.
(DOC)
