# Supplementary material for: Ultrasound Evidence of Early Fetal Growth Restriction after Maternal Malaria Infection
Source: PLoS One. 2012 Feb 9;7(2):e31411. doi: 10.1371/journal.pone.0031411 (PMC3276538; doi:10.1371/journal.pone.0031411)
Supplement: File S1 — Calculations derived from the equations based on a Chinese population. (DOC) [file pone.0031411.s001.doc]

**Supplementary file S1**

Calculations derived from the equations based on a Chinese population [1].

The equations for this population are:

Expected BPD(oi) = −1.295192 + 0.197042 × GA + 0.008247 × GA^2^ − 0.000163 × GA^3^ .

SD = 1.253 × (0.176837 + 0.002714 × GA).

In this equation BPD is in cm and Gestational age (GA) in weeks.

The z-scores were normally distributed. There was a statistically significant difference between the mean (SD) BPD z-scores of the fetuses of infected and uninfected women (0.29 (0.63) versus 0.56 (0.66), p < 0.001) (Table S1). Both *P.falciparum* (or mixed) (n=96) and *P.vivax* (n=240) were associated with a lower z-score than for uninfected women (0.35 (0.69), P=0.004 and 0.26 (0.61), p<0.001, respectively). There was no significant difference in z-score between symptomatic (n=165) and asymptomatic malaria (N=169) episodes, 0.27 (0.63) versus 0.30 (0.64) respectively, p=0.71. Women with multiple malaria infections (n=103) showed the lowest z-scores (0.20 (0.58), P<0.001), but even a single malaria episode (n=233) resulted in a significantly lower BPD z-score: 0.33 (0.65), P<0.001). There was no significant difference in z-score between women with uncomplicated or hyperparasitaemic malaria, but the number of hyperparasitaemic women was too small for further analysis.

References

1. Leung TN, Pang MW, Daljit SS, Leung TY, Poon CF, et al. (2008) Fetal biometry in ethnic Chinese: biparietal diameter, head circumference, abdominal circumference and femur length. Ultrasound Obstet Gynecol 31: 321-327.
