# Supplementary material for: Ultrasound Evidence of Early Fetal Growth Restriction after Maternal Malaria Infection
Source: PLoS One. 2012 Feb 9;7(2):e31411. doi: 10.1371/journal.pone.0031411 (PMC3276538; doi:10.1371/journal.pone.0031411)
Supplement: Table S1 — Factors associated with mean BPD z-score when calculated from the Chinese equation. (DOC) [file pone.0031411.s002.doc]

**Supplementary Table S1.**

Factors associated with mean BPD z-score when calculated from the Chinese equation [1].

|  |  |  | Univariate | | Multivariate (n=2972) | |
| --- | --- | --- | --- | --- | --- | --- |
|  |  | Frequency (%) | Coefficient (95%CI) | P-value | Coefficient (95%CI) | P-value |
| Teenager | No | 3,210 (84.9) |  |  |  |  |
|  | Yes | 569 (15.1) | -0.01 (-0.07, 0.05) | 0.67 | * | NS |
| Primigravidae | No | 2,848 (75.4) |  |  |  |  |
|  | Yes | 931 (24.6) | 0.04 (-0.01, 0.09) | 0.09 | * | NS |
| Smoking | No | 2,893 (76.8) |  |  |  |  |
|  | Yes | 876 (23.2) | 0.02 (-0.03, 0.07) | 0.42 | * | NS |
| Low MUAC | No | 2,801 (96.0) |  |  |  |  |
|  | Yes | 116 (4.0) | 0.04 (-0.09, 0.16) | 0.60 | * | NS |
| Short | No | 2,995 (89.2) |  |  |  |  |
|  | Yes | 362 (10.8) | 0.02 (-0.05, 0.09) | 0.55 | * | NS |
| Low BMI | No | 2,738 (81.6) |  |  |  |  |
|  | Yes | 619 (18.4) | 0.10 (0.05, 0.16) | **<0.001** | 0.09 (0.03 – 0.15) | **0.004** |
| Weight loss | No | 2,396 (80.5) |  |  |  |  |
|  | Yes | 581 (19.5) | 0.05 (-0.01, 0.11) | 0.08 | 0.06 (0.00, 0.12) | **0.04** |
| Anaemia | No | 2152 (56.9) |  |  |  |  |
|  | Yes | 1627 (43.1) | 0.09 (0.05, 0.14) | **<0.001** | $ |  |
| Malaria | No | 3,443 (91.1) |  |  |  |  |
|  | Yes | 336 (8.9) | 0.27 (0.20, 0.35) | **<0.001** | 0.29 (0.210.37) | **<0.001** |
| Symptomatic malaria | No | 169 (50.6) |  |  |  |  |
|  | Yes | 165 (49.4) | 0.03 (-0.11, 0.16) | 0.71 | * | NS |
| Newborn gender | M | 1,936 (51.2) |  |  |  |  |
|  | F | 1,843 (48.8) | 1.42 (0.10, 0.18) | **<0.001** | 0.144 (0.10 – 0.19) | **<0.001** |

Significant differences are shown in bold.

BMI body mass index, MUAC middle upper arm circumference, NS not significant

* Not included in the multivariate analysis

$ Anaemia was highly collinear with malaria, and the co-variates in the multivariate model were unchanged when anaemia was adjusted for or not
